# R/DoctorsUK (Sampling Bias in Action): How English Junior Doctors Pay Referendum Results Compared to Those Predicted on the Doctors UK Reddit

**DOI:** 10.7759/cureus.76714

**Published:** 2025-01-01

**Authors:** Lewis Blenkinsop

**Affiliations:** 1 Surgery, Newcastle Upon Tyne Hospitals NHS Foundation Trust, Newcastle, GBR

**Keywords:** bias, doctorsuk, gmc, pay deal, reddit, resident doctors, social media

## Abstract

Reddit is a popular social media platform that is made up of subreddits, a kind of special interest page. One of these is DoctorsUK, which has over 45,000 members and claims to be a community for UK-based doctors. There is, however, no way of verifying who uses the page, as Reddit is essentially anonymous. Following a period of industrial action in England, a pay deal was put forward and voted on by over 45,000 resident doctors in England. During the voting window, many posts on the subreddit DoctorsUK referenced the pay deal with various comments stating that members were either voting for or against the deal. I wanted to compare the numbers who stated their views on the page to the results obtained in the official ballot. I analyzed the four most commented - on posts relating to the pay deal and read a total of 1,297 comments on these posts. Only a user’s first comment on a post was included in the analysis, and deleted comments were not retrieved. This left me with 548 comments to include in the study. I then grouped the comments based on whether they clearly stated their voting intention on the ballot. The voting intention was clearly stated in 230 comments. Of these 230 comments, 60 comments (26.1%) said they would be accepting the deal, and 170 (73.9%) said they would be rejecting it. In the formal ballot, 66% of resident doctors voted to accept the deal. A sentiment analysis was also performed, which found an overwhelming negative sentiment in three of the four posts. A chi-squared test found a P-value of <0.001, suggesting a significant difference between predicted and actual values. While this paper looked at a single question, it found a significant amount of bias in the subreddit. There are few surveys of doctors at such volume that other issues could be assessed in this way. This study should lead to a questioning of the veracity of views on a page that is anonymous and a realisation that we can, not in good faith, assume that all posters on DoctorsUK are UK-based doctors. With this in mind, we may need to question the possible alternative motives for the propagation of such ideas. It is also worth considering how these ideas being shared in a public forum associated with the medical profession may affect large language models and AI, given that Reddit has allowed AI companies access to its data.

## Introduction

Reddit is an online social media platform with over 300 million weekly users, a number growing by more than 50% year on year [1]. Users on Reddit do not use their real names, but it is against the rules to impersonate an individual or entity in a misleading or deceptive manner [2]. Subreddits are pages on Reddit dedicated to one particular topic. The Doctors UK subreddit describes itself as “A community for UK-based doctors to chat about their experiences, share articles and hang out. Medical advice is not to be sought here” and has over 45 thousand members. Its subreddit has rules including “Avoid excessive negativity” and “No medical queries” [3]. The page was initially created in October of 2021 and is a forum for sharing experiences from users claiming to be UK doctors, but their identities are anonymous. Posts on any subreddit can receive comments. These comments can then be sorted based on the time they were posted or depending on the number of “upvotes” or “downvotes” they receive. The “top comments” are defined as the ones with the highest number of “upvotes” after the “downvotes” have been subtracted.

The GMC defines social media as “the use of private messaging, websites, and applications that enable users to create and share content or to participate in social networking” [4]. They also say, “It is important that…people who access what you say about health and healthcare have information that supports their understanding and helps them to verify your claims and expertise. If you’re commenting on health or healthcare issues, you should usually say who you are”[4]. Based on these assessments it would appear that the Doctors UK subreddit is an example of social media and that the majority of posts do not comply with the requirement to say who you are, given the majority of Reddit users do not use their real names.

Artificial intelligence (AI) large language models require large volumes of text to analyse to understand and learn how human conversation takes place. Multiple court cases have arisen due to this requirement, with AI companies accused of using data such as news articles without permission. The AI companies may face heavy fines if they are found guilty [5,6]. In response to this, AI companies and Reddit have agreed on a deal to sell the information on subreddits to help AI large language models [7,8].

On 16/09/2024, Resident Doctors in England voted to accept a pay deal proposed by the government following multiple rounds of industrial action. A total of 45,830 doctors voted in the pay deal referendum(a turnout of 69% of eligible union members) of these 30,227 (66%) voted in favour of the deal [9]. From the time the deal was initially sent to members to vote on, to the time that the results were released, various posts were shared on the Doctors UK subreddit relating to the proposed deal. As with other posts on the subreddit, the commenters tended to be anonymous and claimed to be doctors in the UK who would be voting on the deal.

The aim of this study is to assess how accurately the views expressed on the Reddit page represent the Resident Doctor community in the UK. To do this I would look at the most commented upon posts relating to the pay deal and analyse the comments below these posts, I will then compare these comments to the outcome of the pay referendum. I have chosen the pay deal as the focus of the study as there are few ballots of doctors that receive as high a turnout as the pay deal and few other ballots that ensure those voting are medical professionals. I will go on to discuss the implications of any bias on public perception and AI. One clear drawback of this study is that the page is for Doctors throughout the UK, while the ballot measure was only for Resident Doctors in England. Another limitation is that the study will only look at the single question of accepting or rejecting the pay deal and how representative the page is on this issue.

## Materials and methods

A search was performed on the Doctors UK Subreddit on 29/11/2024. The search terms were “pay deal” and “pay offer”. The four posts with the most comments between these two search terms were then analysed. On these posts, every comment and every reply to a comment was reviewed. This meant a total of 1,297 comments were reviewed over the four posts.

The four most commented-upon posts were as follows: “Junior doctors offered 20% pay rise to end strike actions” [10] with 510 comments; “Pay deal accepted!” [11] with 452 comments; “[Summary] Arguments around the Pay Offer” [12] with 169 comments; and “The deal we are voting on is 4%. We already have the 17% increase banked with back pay. Even if we vote No” [13] with 166 comments. Every comment thread was expanded to read the full discussion related to each comment.

We excluded comments if the same user posted multiple replies under the same post, only the users' first comment was analysed, and subsequent ones were ignored. Deleted comments were not recovered. After repeated comments and deleted comments were removed, there was a total of 548 comments over the four posts included in the study.

Comments were graded as either accept, reject, or unclear. Comments were only classified as accept or reject if that was explicitly stated as the commenter's referendum voting intention. If the comment seemed negative but did not explicitly say they were going to reject the offer, then it was classed as unclear. The same was true of positive comments. If there was any uncertainty about the commenters' voting intention, it was listed as unclear.

The “Top” three comments on each post were separately documented as accept, reject, or unclear. This was performed to gauge the most popular sentiment on each post. I also instructed ChatGPT to conduct sentiment analysis on each of the posts and their associated comments [14] following the extraction of the Reddit page into a PDF, the full analysis being present in the appendixes of this paper. This provided an idea of the sentiment of all of the comments on the post as, once again, all comments and replies had been expanded.

A goodness of fit chi-squared test was also performed on the subsequent total comments accepting or rejecting the deal. This was performed by comparing the observed values, those present on the Reddit page, to the expected values, the results of the pay referendum. If the Reddit page were representative of the issue of the pay deal, we would expect a higher p-value and acceptance of the null hypothesis that the voting intention expressed on the Reddit page is representative of the ballot outcomes.

## Results

“Junior doctors offered 20% pay rise to end strike actions” [10] had a total of 510 comments on the post. As described in the method section, repeated comments and deleted comments were removed from this total, leaving us with 272 comments included in the study. The breakdown of these comments is presented in Table 1. Of these 272 comments, 185 (68%) did not clearly express their voting intention. Of the remaining 87 comments that clearly expressed their intention, 31 comments (35.6%) said they would be accepting the offer, and 56 comments (64.4%) said they would be rejecting it. Of the top three comments on this post, one stated they would reject the offer, while the other two were unclear. The sentiment analysis from ChatGPT found “General Tone: The comments reflect a mix of frustration, skepticism, cautious optimism, and dissatisfaction, with a leaning towards negative sentiment overall due to concerns about fairness, inflation and comparison with other professions.” (Appendix 1) [14].

**Table 1:** Analysis of comments on the post "Junior doctors offered 20% pay rise to end strike action"

Junior doctors offered 20% pay rise to end strike actions	Comments that were unclear	Comments that stated they would accept	Comments that stated they would reject
Number of comments	185	31	56
Percentage of comments	68.0%	11.4%	20.6%
Percentage of Accept/Reject	N/A	35.6%	64.4%

“Pay deal accepted!” [11] had a total of 452 comments on the post. Of these, 140 were included in the study. The breakdown of these comments is presented in Table 2. Of the 140 comments, 71 comments (50.7%) did not clearly express their voting intention. Of the remaining 69 comments that clearly expressed their intention, 15 comments (21.7%) said they would be accepting the offer, while 54 comments (78.3%) said they would be rejecting it. Of the top three comments on this post, two said that they would be rejecting the offer, while one was unclear. The sentiment analysis from ChatGPT found “The majority of the comments express negative or mixed sentiments, reflecting dissatisfaction, frustration, and cautious acceptance. A minority of comments display positive sentiments, showing support for moving forward despite the deal's limitations.” (Appendix 2) [14].

**Table 2:** Analysis of comments on the post "Pay deal accepted!"

Pay deal accepted!	Comments that were unclear	Comments that stated they would accept	Comments that stated they would reject
Number of comments	71	15	54
Percentage of comments	50.7%	10.7%	38.6%
Percentage of Accept/Reject	N/A	21.7%	78.3%

“[Summary] Arguments around the Pay Offer” [12] had a total of 169 comments on the post. Of these, 90 were included in the study. The breakdown of these comments is presented in Table 3. Of the 90 comments, 33 comments (36.7%) did not clearly express their voting intention. Of the remaining 57 comments that clearly expressed their intention, eight comments (14%) said that they would be accepting the offer, while 49 comments (86%) said that they would be rejecting it. Of the top three comments on this post, all three said they would be rejecting the offer. The sentiment analysis from ChatGPT found “Overall Sentiment: Negative. The tone across the document is predominantly critical and dissatisfied. Key themes include: The inadequacy of the pay offer relative to inflation and historic pay erosion. Perceived lack of commitment to full pay restoration (FPR). Criticism of the government’s and BMA’s approaches, including strategic concerns.” (Appendix 3) [14].

**Table 3:** Analysis of comments on the post "[Summary] Arguments around the pay offer"

[Summary] Arguments around the Pay Offer	Comments that were unclear	Comments that stated they would accept	Comments that stated they would reject
Number of comments	33	8	49
Percentage of comments	36.7%	8.9%	54.4%
Percentage of Accept/Reject	N/A	14.0%	86.0%

“The deal we are voting on is 4%. We already have the 17% increase banked with back pay. Even if we vote No” [13] had a total of 166 comments on the post. Of these, 46 were included in the study. The breakdown of these comments is presented in Table 4. Of the 46 comments, 29 comments (63%) did not clearly express their voting intention. Of the remaining 17 comments that clearly expressed their intention, six comments (35.3%) said that they would accept the offer, while 11 comments (64.7%) said that they would reject it. Of the top three comments on this post, one said it would be accepting the deal, one said it would be rejecting it, and one was unclear. The sentiment analysis from ChatGPT found “Overall Sentiment: Mixed. While there are negative sentiments about the inadequacy of the pay offer and the comparison to Physician Associate salaries, there are also more pragmatic and supportive views expressed regarding the deal as a step forward.” (Appendix 4) [14].

**Table 4:** Analysis of comments on the post "The deal we are voting on is 4%..."

The deal we are voting on is 4%. We already have the 17% increase…	Comments that were unclear	Comments that stated they would accept	Comments that stated they would reject
Number of comments	29	6	11
Percentage of comments	63.0%	13.0%	23.9%
Percentage of Accept/Reject	N/A	35.3%	64.7%

Over the four posts, 548 comments were included in the study. The cumulative breakdown of these comments is presented in Table 5. Of the comments, 318 comments (58%) did not clearly express their voting intention. Of the remaining 230 comments that clearly expressed their intention, 60 comments (26.1%) said that they would accept the offer, while 170 comments (73.9%) said that they would reject it.

**Table 5:** Analysis of the total comment numbers over the four posts

Total comments over four posts	Unclear	Accept	Reject
Number of comments	318	60	170
Percentage of comments	58.0%	11.0%	21.0%
Percentage of Accept/Reject	N/A	26.1%	73.9%

This gives the final results of 60 comments for accept (26.1%) and 170 comments for reject (73.9%) compared to the ballot results [9] of 66% accept and 34% reject. If the Reddit posts had been representative of the ballot outcome, we would have expected 151.7 accepts and 78.3 rejects.

A chi-squared test was then performed on these data with the null hypothesis that the voting intention expressed on the Reddit page is representative of the ballot outcomes. The expected values of 151.7 accept and 78.3 rejections were input along with the observed values of 60 accepts and 170 rejects. This gives us a chi-squared value of 162.8 with one degree of freedom. This results in a p-value of 2.73×10−37. This means that the null hypothesis is rejected. This shows that there was a significant difference between the views expressed on the Reddit page and the ballot result.

## Discussion

This study shows that, during the industrial action and subsequent pay deal negotiations in the summer of 2024, the Reddit page “Doctors UK” was not representative of the voting intentions of resident doctors in England. Further specific conclusions are limited. The bias observed may be an example of sampling error, with only 230 comments included compared to 45,830 votes cast in the pay deal referendum [9]. The 230 comments may have all been posted by resident doctors eligible to vote in the referendum, but due to Reddit's anonymous profiles, it is impossible to confirm this.

I previously mentioned the General Medical Council (GMC) guidance on identifying yourself when using social media [4]. This guidance is designed to benefit patients so they know who is giving advice or opinions in online forums. This study demonstrates the benefit of this rule for other doctors. There is no way of knowing who comments on the Reddit page; they could be a doctor, a recruiter, or a disgruntled patient. Medical school is difficult to enter, with competition ratios between 5:1 and 18:1 depending on the university [15]; due to this, you can assume strong candidates enter medical school and that graduates would be highly sought after in other fields. It is known that, in the world of business, doctors can pursue alternative careers [16], and if doctors want to continue working in healthcare, the appeal of working abroad has rarely been stronger, with up to one in three doctors considering working abroad at some point [17]. My concern is that, with the anonymity afforded by Reddit, there would be nothing to stop recruiters from business or overseas healthcare agencies from propagating negative views and stories of UK healthcare. Spreading these views could help recruit medics for other jobs in the private industry or abroad. There would also be no way of proving this was happening. The sentiment analysis on the four posts showed that the overwhelming emotion shared in the comments was negative. This could be an honest representation of some doctors, or it could be misleading posts by people with ulterior motives. It is known that negative posts are more likely to be shared, and given how top comments are calculated on Reddit, it may incentivize negativity. This, however, would not explain the skew in voting intention, as the same research finds people in general are more likely to post positive content. It is worth noting, however, that this research looked at Twitter rather than Reddit, and the anonymity of Reddit may further skew the positive/negative share of comments [18,19].

Another concern is regarding the use of Reddit pages to train large language models. On a page reportedly of doctors in the UK, there is an overwhelming negative sentiment in the four posts reviewed. In addition to this, the views were not representative of the actual body the page was meant to represent, as evidenced by this study. If an AI program learns to relate the opinions on the page with those of doctors in the UK, I worry about how it may present this information and represent resident doctors as a body. I do not know what conclusions it would draw, but based on this study, those conclusions may not be representative of the actual profession.

My final concern relates to it being a public forum. Anybody with an internet connection can view the page and read the apparent opinions of hundreds of unverified doctors in the UK. The topics shared on the page vary widely and may cause upset among the general public. However, it is not just the public viewing the page; there are a variety of newspaper articles that explicitly reference this Reddit forum as a source for the view of doctors. This does not even begin to consider the stories that do not explicitly quote it as a source but may consider it as a starting place for journalistic ideas [20,21]. With no way of confirming the identity of the poster and with a post made on a page that is not always representative of doctors, I worry that articles could be written and that the Reddit page may be listed incorrectly as a source for doctors' viewpoints. It is also worth noting that doctors themselves are not best placed to assess public opinion and that the public may feel posts are unprofessional or breach confidentiality more often than a doctor would themselves [22].

There are various limitations to this study. The study only looks at a single issue and how representative the page was on this single issue. This is due to the limited number of large-scale ballots conducted on doctors. It was only able to look at a minority of the comments, as only a minority expressly stated their voting intentions. The comments analysed were by definition qualitative data, and in trying to extract quantitative information from it, I was limited in which comments could be included. Originally, I had hoped to work with a colleague to assign a score to each comment to grade how “for” or “against” the deal they were. Unfortunately, when we started with this method, we found that, with the majority of comments, it was impossible to discern for sure whether the commenter was against the pay offer or against other people's comments on the post. There is also a subculture within Reddit, including the use of acronyms and inside jokes that I worried may make analysis by a large language model limited, so I proceeded only with a sentiment analysis from ChatGPT. Because of these limitations, we proceeded with binary results, only including comments if the commenter had clearly stated their voting intention.

## Conclusions

This study has demonstrated that the Reddit page Doctors UK was not representative of the resident doctor body in England during the pay deal negotiations and subsequent referendum in the summer of 2024. The page had a significantly higher percentage of users in favor of rejecting the deal than in the referendum, 73.9% on the page vs. 34% on the ballot. The comments of people saying they would reject the deal were often the “top comments” under posts relating to the pay deal and industrial action.

There is no way of verifying the identities of the Reddit users who post on the page. Therefore, the page should not be considered a reliable tool for gauging the views of UK doctors.

## Appendices

**Table 6:** Appendix - Full sentiment analysis of posts and comments

Appendices	
Appendix 1	Here’s a detailed sentiment analysis of the document based on the comments: Overall Sentiment General Tone: The comments reflect a mix of frustration, skepticism, cautious optimism, and dissatisfaction, with a leaning towards negative sentiment overall due to concerns about fairness, inflation, and comparison with other professions. Detailed Breakdown Negative Sentiment: Reasons: Many comments express frustration about the offer being inadequate in addressing inflation and pay erosion over time. Comparison of junior doctors' pay with physician associates (PAs) and other healthcare staff highlights perceived unfairness. Concerns about long-term implications, lack of inflation linkage, and perceived "spin" in marketing the pay rise as 20%. Example Phrases: “Nowhere near good enough.” “This is a very bad deal.” “Absolutely disgusting. Can’t wait to leave this country.” “Fat no from me!” Positive Sentiment: Reasons: Some comments recognize the offer as a step forward compared to past negotiations. Acknowledgment of improvements in pay backdating and above-inflation raises for a couple of years. Example Phrases: “This is better than expected.” “It’s a promising move in the right direction.” “Take the win.” Neutral or Mixed Sentiment: Reasons: Many comments analyze the situation with calculations, discussions of broader economic factors, and strategic recommendations. Some reflect pragmatic views about accepting the offer now and pushing for further improvements later. Example Phrases: “It’s a compromise.” “Bank it and strike again in the future.” “This is a great first step.” Quantitative Estimate: Out of all the comments: 60% Negative 20% Positive 20% Neutral/Mixed Key Themes Identified: Disparity in Pay: Strong dissatisfaction about PAs earning more than doctors. Inflation Adjustment: Repeated calls for pay linked to inflation to prevent future erosion. Skepticism Towards Marketing: Comments critical of the government and media’s portrayal of the pay rise as generous. Pragmatism and Strategy: Suggestions to accept the offer as a short-term win while preparing for future strikes. If you’d like, I can visualize this sentiment analysis (e.g., a pie chart) or provide insights into specific aspects. Let me know!
Appendix 2	The document contains comments from a Reddit thread discussing the acceptance of a pay deal for doctors in the UK. Below is a sentiment analysis summary: Overall Sentiment The majority of the comments express negative or mixed sentiments, reflecting dissatisfaction, frustration, and cautious acceptance. A minority of comments display positive sentiments, showing support for moving forward despite the deal's limitations. Sentiment Breakdown Negative Sentiments: Themes: Frustration with the deal, lack of pay restoration (FPR), low morale, and disappointment in the profession's collective stance. Examples: "Is it any surprise UK medicine is a complete shambles when a majority of doctors roll over when the government throws them a bone?" "This is a pitiful offer but worryingly only a 69% turnout." Mixed Sentiments: Themes: Reluctance to accept the deal but acknowledging pragmatic reasons, concerns about future negotiations and strikes. Examples: "Even though I voted yes in the end it was a reluctant yes on the basis that our negotiating committee is changing and infighting in the ranks in the BMA." "I voted no. My wife, my friends, my senior colleagues all voted yes. So this result is expected." Positive Sentiments: Themes: Support for moving forward, optimism about future negotiations, and gratitude for progress made. Examples: "We the 34 percent are always here for action!" "I am fully on board and willing to go on strike again in April around the next DDRB, and I think many doctors will be." Themes Identified Pay Parity Concerns: Comparisons between doctors’ pay and that of physician associates (PAs) generated strong dissatisfaction. Strike Sentiment: Divisions over whether the deal justifies continued industrial action. Future Prospects: Skepticism about achieving FPR and concerns about diminishing enthusiasm for strikes. Cautious Acceptance: Some view the deal as a temporary measure and advocate for continued pressure. Would you like a deeper dive into specific themes, or analysis of individual comments?
Appendix 3	To perform a sentiment analysis on the entire document, I will analyze the tone and sentiment of the key arguments and statements provided. Here's the sentiment breakdown: Sentiment Analysis Results Overall Sentiment: Negative The tone across the document is predominantly critical and dissatisfied. Key themes include: The inadequacy of the pay offer relative to inflation and historic pay erosion. Perceived lack of commitment to full pay restoration (FPR). Criticism of the government’s and BMA’s approaches, including strategic concerns. Key Sentiment Highlights Pay Offer (Negative): The offer is described as insufficient, merely reverting pay to 2020/2021 levels and failing to address future inflation. Sentiment is compounded by comparisons where junior doctors’ salaries are highlighted as being below those of physician associates (PAs), fostering frustration and indignation. BMA's Position (Skeptical/Negative): The BMA’s lack of enthusiasm for the offer and the perceived lack of advocacy for full pay restoration is criticized. The sentiment indicates distrust in the process, with suggestions that the BMA was pressured into presenting the offer. Public Opinion and Government Strategy (Critical/Negative): The government’s tactics, such as leaking the offer to media ahead of the BMA announcement, are seen as undermining trust. The document stresses the importance of prioritizing junior doctors’ needs over public opinion. Future Strikes and Negotiations (Pessimistic/Negative): Accepting the offer is viewed as weakening bargaining power and discouraging future industrial action. Long-term strategic concerns dominate, with warnings against repeating past mistakes. General Conclusion (Resolute but Negative): The document strongly advocates rejecting the offer, emphasizing it as inadequate and unaligned with members' demands for pay restoration. The overall sentiment reflects dissatisfaction with the pay offer, skepticism toward leadership decisions, and determination to seek better terms. If you'd like more detailed insights or additional breakdowns, let me know!
Appendix 4	The second document provides arguments and perspectives regarding voting on a proposed 4% pay increase for UK doctors, following a 17% increase already received with back pay. The tone is mixed but includes strong opinions both in favor of and against the pay deal. Sentiment Analysis Results Overall Sentiment: Mixed While there are negative sentiments about the inadequacy of the pay offer and the comparison to Physician Associate salaries, there are also more pragmatic and supportive views expressed regarding the deal as a step forward. Key Sentiment Themes Criticism of the Pay Offer (Negative): Many express dissatisfaction with the additional 4% pay rise, viewing it as insufficient compared to inflation and historical pay restoration goals. Frustration is noted over junior doctors being paid less than Physician Associates, even with the proposed increase. Support for Acceptance (Positive/Pragmatic): Some view the pay offer pragmatically, highlighting that 17% has already been secured and advocating for acceptance of the 4% as part of ongoing progress. A sentiment of trust in the negotiation process is also present, with some arguing that a "No" vote could weaken the union's position for future negotiations. Long-Term Perspective (Neutral to Negative): Concern is expressed about the long-term implications of rejecting the deal, including the risk of stalled progress and further strikes. Calls for unity among doctors emphasize the need for solidarity, regardless of voting stance, to avoid divisive rhetoric. Emotional Reactions (Polarized): Some argue emotionally for rejecting the deal, emphasizing a commitment to further strikes for full pay restoration. Others criticize what they perceive as an overly idealistic or divisive stance, urging practical compromise. If you'd like a deeper dive into specific arguments or sentiments, let me know!
